# Diagnostic indices for vertiginous diseases

**DOI:** 10.1186/1471-2377-10-98

**Published:** 2010-10-25

**Authors:** Otmar Bayer, Jan-Christian Warninghoff, Andreas Straube

**Affiliations:** 1 Integrated center for research and treatment of vertigo, balance and ocular motor disorders Ludwig-Maximilians-University of Munich, Heiglhofstr. 63, 81377 Munich, Germany; 2 Isar-Amper-Klinikum München-Ost, Vockestraße 72, 85540 Haar bei München, Germany; 3 Department of Neurology, University of Munich, Marchioninistraße 15, 81377 Munich, Germany

## Abstract

**Background:**

Vertigo and dizziness are symptoms which are reported frequently in clinical practice. We aimed to develop diagnostic indices for four prevalent vertiginous diseases: benign paroxysmal positional vertigo (BPPV), Menière's disease (MD), vestibular migraine (VM), and phobic postural vertigo (PPV).

**Methods:**

Based on a detailed questionnaire handed out to consecutive patients presenting for the first time in our dizziness clinic we preselected a set of seven questions with desirable diagnostic properties when compared with the final diagnosis after medical workup. Using exact logistic regression analysis diagnostic scores, each comprising of four to six items that can simply be added up, were built for each of the four diagnoses.

**Results:**

Of 193 patients 131 questionnaires were left after excluding those with missing consent or data. Applying the suggested cut-off points, sensitivity and specificity were 87.5 and 93.5% for BPPV, 100 and 87.4% for MD, 92.3 and 83.7% for VM, 73.7 and 84.1% for PPV, respectively. By changing the cut-off points sensitivity and specificity can be adjusted to meet diagnostic needs.

**Conclusions:**

The diagnostic indices showed promising diagnostic properties. Once further validated, they could provide an ease to use and yet flexible tool for screening vertigo in clinical practice and epidemiological research.

## Background

Vertigo and dizziness are, like headache very prevalent symptoms in daily clinical practice. The life time prevalence is estimated to be 20 - 30% [1]. For the symptom headache it was shown that a very simple screener with only three questions are able to differentiate headaches with a sensitivity of 0.81 (95% CI 0.77 to 0.85), a specificity of 0.75 (95% CI 0.64 to 0.84), and a positive predictive value of 0.93 (95% CI, 89.9 to 95.8) to predict a migraine [2]. Therefore we investigated whether such a screener which can be easily filled out by the patients during the time in the waiting room can be also developed for patients suffering from vertigo or dizziness. We focused our efforts on the differentiation of the most prevalent diagnoses benign paroxysmal positional vertigo (BPPV), Meniere's disease (MD), vestibular migraine (VM) and phobic postural vertigo (PPV) since these four diagnoses cover about 54% of all patients in a dizziness out patient unit [1]. The screener was developed by analysing a larger questionnaire, which was administered to patients presenting in a dizziness clinic at the neurology department of Munich university, a tertiary center for vertigo disorders.

## Methods

We conceived a short questionnaire by analysing and subsequently condensing a detailed questionnaire designed for patients suffering from vertiginous diseases. The detailed questionnaire with specific questions about vertiginous diseases evolved on the basis of the pain questionnaire of the German Society for the Study of Pain http://www.dgss.org, chapter of the IASP. Data collection was done between 2003 and 2007. In order to get detailed and structured information about the history of the patients and the signs of the actual clinical symptoms we asked the patients to fill in the questionnaire. Since this data collection was introduced as a pilot the questionnaire was handed out on predefined dates (usually once a week) to all patients presenting for the first time in the dizziness clinic on that day to obtain an unbiased sample. All patients gave their written informed consent to this procedure. Since the study was not experimental, and the data were gained in clinical routine, the approval of an ethics committee was not necessary. All data were anonymised. All patients were seen by two experienced neurologists who were blinded to the answers given in the questionnaire and received a complete medical work up with patients undergoing a clinical neurological examination, orthoptic examination, eye movement recording, and, if necessary, Doppler sonography of the cranial vessels, evoked potentials, cranial imaging and consultations of other specialities (e.g. ENT, ophthalmology, and psychiatry). For further details see [3]; the questions are listed in the additional file 1.

The analyses were based on clinical diagnoses rather than on restrictive inclusion criteria, such as used e. g. in clinical trials. Although the latter approach has the advantage of high diagnostic accuracy, it restricts the study sample to typical patients with clear syndromes, which does not always match with clinical reality. Diagnostic criteria applied in the clinic were: for PPV as described by Brandt [4]. BPPV was diagnosed if reproducable by positioning maneuvers, or in case of a distinctive history with other causes ruled out. MD according to the AAO-HNS [5,6] criteria, if hearing loss was not audiologically documented before or in the ENT department, anamnestic hearing loss was accepted. VM patients fulfilled the criteria of definite or probable migrainous vertigo [7].

We developed diagnostic indices for the four most frequent diagnoses: PPV (n = 53), BPPV (n = 19), VM (n = 14) and MD (n = 11). First we screened the detailed questionnaire for items potentially useful for diagnostic indices:

◦ The kind of vertigo (rotational vertigo, unsteadiness, feeling of being in a lift, lightheadedness)

◦ Perception of the environment (like on a roundabout, like on a boat, very blurred)

◦ The occurence of vertigo (in attacks, persistent, persistent with attacks)

◦ Duration of attacks (seconds, minutes, hours, days, more than one week)

◦ Intensity of vertigo attacks and intensity of persistent vertigo (intensity scale from no vertigo "0" to the most intense possible vertigo "10")

◦ Trigger with pre-formulated answers: "alleviating", "no influence" or " amplifying" (physical load, psychological load, darkness or bad sight, turning while staying in bed, head inclination, bending down, raise, relaxing itself, shaking the head, cough, large heights)

◦ Concomitant symptoms with pre-formulated answers: "always", "frequently", "occasionally", "never" (vision disorders, diplopic images, speech disorder or dysphagia, paraesthesia, paralysis, sweating, drop seizure, headache, defective hearing, ear noises, nausea, vomiting, impaired consciousness)

After screening of the larger questionnaire the following items were chosen for further investigation: The kind of vertigo (rotational vertigo, unsteadiness, feeling of being in a lift, lightheadedness), perception of the environment (like on a roundabout, like on a boat, very blurred), and the concomitant symptoms defective hearing, ear noises, nausea, vomiting, sweating, drop seizures with pre-formulated answers "always", "frequently", "occasionally", "never".

Sensitivity, specificity, positive and negative predictive value with respect to the four main diagnoses were calculated for these questions (Table 1). Based on the positive likelihood ratio (i. e. sensitivity/(1 - specificity), primary criterion) and the other test measures mentioned above, variables were built and preselected as candidates to be included in the diagnostic score. To build the diagnostic score multivariate logistic regression modelling was applied using a backward elimination strategy for variable selection. The full model included all preselected variables. The effect estimates with the highest p-values were identified and the corresponding variables were removed successively until only effects significantly differing from 0 with p < 0.20 were left in the model. The linear predictor of the resulting final model was used as the diagnostic score, and calculated for each patient with sufficient data. Finally, receiver operating characteristics (ROC) curves were drawn, where the area under the curve (AUC) served as a measure of the diagnostic index's test power. An AUC of 1 indicates a perfect test.

**Table 1:** Sensitivity and specificity of the items included in the diagnostic indices.

	PPV		BPPV		MD		VM	
	
	**sens**.	**spec**.	**sens**.	**spec**.	**sens**.	**spec**.	**sens**.	**spec**.
**occurrence of vertigo**								

in attacks	0.32	0.28	0.74	0.47	0.91	0.48	0.79	0.47

as persistent v~	0.38	0.91	0.00	0.76	0.00	0.78	0.00	0.77

**kind of vertigo**								

rotatory vertigo	0.21	0.25	0.79	0.62	0.64	0.57	0.79	0.60

unsteadiness	0.64	0.68	0.16	0.43	0.46	0.51	0.43	0.50

lift feeling	0.15	1.00	0.00	0.89	0.00	0.91	0.00	0.90

lightheadedness	0.40	0.79	0.11	0.64	0.09	0.66	0.43	0.71

**perception of environment**								

like on a roundabout	0.26	0.26	0.79	0.58	0.64	0.51	0.77	0.54

like on a boat	0.64	0.65	0.32	0.46	0.46	0.50	0.31	0.47

very blurred	0.21	0.98	0.00	0.85	0.00	0.87	0.08	0.88

**ear noises**								

never - occasionally	0.70	0.33	0.88	0.35	0.10	0.23	0.86	0.34

frequently - always	0.30	0.68	0.12	0.66	0.90	0.75	0.14	0.62

**defective hearing**								

never - occasionally	0.89	0.30	0.94	0.23	0.10	0.11	0.86	0.21

frequently - always	0.11	0.68	0.06	0.77	0.90	0.89	0.14	0.65

**nausea/vomiting/sweating**								

never - occasionally	0.65	0.59	0.78	0.52	0.20	0.41	0.07	0.38

frequent - always	0.35	0.40	0.22	0.48	0.80	0.58	0.93	0.62

**drop seizure**								

never - occasionally	0.80	0.24	1.00	0.27	0.50	0.23	0.64	0.19

frequently - always	0.20	0.76	0.00	0.73	0.50	0.82	0.36	0.81

Note that the cut-off points for the ordinal variables ear noises, defective hearing, nausea/vomiting/sweating, and drop seizures were chosen arbitrarily and do not necessarily match those of the diagnostic indices.

The whole selection and modeling procedure was done separately for the four diagnoses. To address the problem of collinearity in multivariate modelling, we computed Spearman's rank correlation coefficients (Table S1 in additional file 1) supplemented by a priori knowledge to identify prediction variables of similar content (rho >= 0.5). Whenever two such variables (e.g. rotational vertigo and like on a roundabout) appear in a model, the effect estimates are likely to become insignificant. In these situations we tried replacing the variables by one indicating, if at least one of the underlying questions was answered positively; in case of the example rotational vertigo or like on a roundabout. In cases of doubt we favoured the model resulting in the better AUC. A useful side effect is that this approach also increases the proportion of evaluable scores in case of incompletely filled in questionnaires.

Due to a limited number of cases for some diagnoses, some cells in the contingency tables happened to be empty (e. g. none of the MD patients indicated having persistent vertigo). This causes the problem of quasi-complete separation of the data, which corrupts the corresponding maximum likelihood estimates in the usual logistic regression. This problem is commonly circumvented by excluding the respective variables. However, when building diagnostic indices, such a procedure could lead to the exclusion of variables with high sensitivity or specificity. We therefore applied exact logistic regression using Firth's 2nd order bias correction [8-10], a method, which is now available in major statistical software packages, that has been demonstrated to give proper results in the situation just described [11].

## Results

Of 193 patients 131 (74 female and 57 male, mean age 54, ranging from 16 to 90 years) were included, while the remaining 62 patients were excluded because of missing data or missing consent for this observational study.

### Diagnostic index for PPV

**Figure 1:**
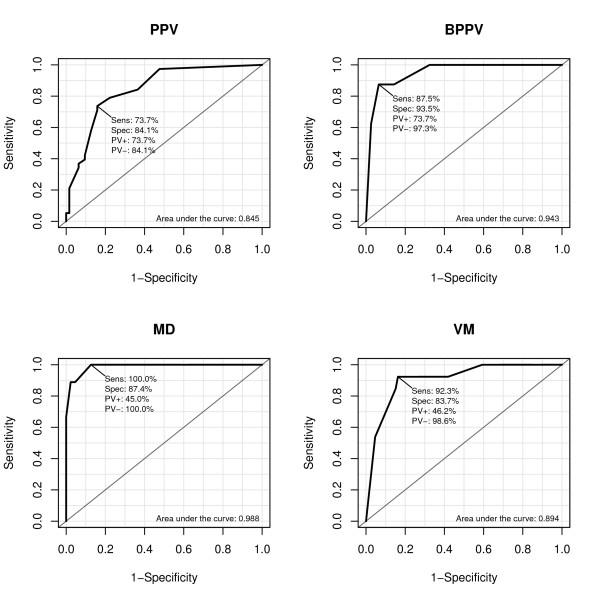
**ROC curves of the diagnostic score for phobic postural vertigo (PPV), benign paroxysmal positional vertigo (BPPV), Menières disease (MD), and vestibular migraine (VM)**. Abbreviations used in the figure: Sensitivity (Sens), Specificity (Spec), positive predictive value (PV+), negative predictive value (PV-).

After the selection process described in methods, the calculation of the diagnostic score for PPV was reduced to five items as detailed in Table 2. When a cut-off point of 0.31 is used, this diagnostic index had a sensitivity of 73.7% and a specificity of 84.1%. The ROC curve in Figure 1 depicts other sensitivities/specificities that can be obtained by using other cut-off points. If, for example, a very high sensitivity of 0.97 is desired, the specificity would be lowered to 1 - 0.48 = 0.52. The area under the curve was 0.845.

**Table 2:** Calculation of the diagnostic scores for PPV, BPPV, MD, and VM.

Question - Item/answer	PPV	BPPV	MD	VM
How does your vertigo occur?				
in attacks	0		3.77	
as persistent vertigo	2.22	-2.35		-1.79
as persistent vertigo with attacks	1.65			

What kind of your vertigo do you have?				
rotational vertigo	-1.48^1^			1.55^1^
unsteadiness		-3.26		
feeling of being in a lift	1.79	-3.06		
lightheadedness			-2.54	

How do you perceive the environment during vertigo?				
like on a roundabout	-1.48^1^			1.55^1^
like on a boat				
blurred		-3.42	-3.74	

How often do you have defective hearing?				
never				
occasionally				-1.14^2^
frequently				-1.14^2^
always				-1.14^2^

How often do you have ear noises?				
never				
occasionally				-1.14^2^
frequently				-1.14^2^
always		-3.03	5.42	-1.14^2^

How often do you have sweating/nausea/vomiting?				
never			0 = 0 · 0.98	
occasionally			0.98 = 1 · 0.98	
frequently		-1.21^1^	1.95 = 2 · 0.98	2.82
always		-1.21^1^	2.93 = 3 · 0.98	

How often do you have drop seizures?				
never				
occasionally				
frequently	1.70	-2.95^2^		
always		-2.95^2^		

For answers marked in the questionnaire, the corresponding numbers are added up. Items marked with the same superscript number within one diagnosis are scored only once. E. g. the score for PPV of a patient reporting „rotational vertigo“ or „perception of the environment like on a roundabout“ (both marked by 1) will be diminished by 1.48 no matter whether this patient reports both or only one of these two symptoms. Patients are allowed to check only one answer per question, except for the 2nd and 3rd question.

Examples of calculated scores can be found in the Additional file 1

### Diagnostic index for BPPV

The diagnostic index for BPPV used two more items; the AUC of the ROC was 0.943. Using -1.22 as the cut-off point resulted in a sensitivity of 87.5%, specificity of 93.5% and a positive predictive value of 73.7% (Figure 1).

### Diagnostic index for MD

The diagnosis of MD was predicted by vertigo appearing in attacks, the kind of vertigo, the perception of the environment as well as the concomitant symptoms ear noises and nausea, sweating, vomiting. The odds of the outcome MD were associated with an increasing frequency of these vegetative symptoms in a quite loglinear fashion. At a cut-off point of 6.70 a sensitivity of 100% and a specificity of 87.4% were computed for this diagnostic index, while the AUC was 0.988 (Figure 1).

### Diagnostic index for VM

The diagnostic index for VM contained persistent vertigo, rotational vertigo or perception of the environment like on a roundabout, defective hearing or ear noises, and sweating/nausea/vomiting. Using a cut-off point of 2.81 resulted in a sensitivity of 92.3% and specificity of 83.7%, the AUC was 0.894 (Figure 1).

## Discussion

The diagnostic indices were developed as an instrument to preselect patients with vertiginous diseases using a simple screener on the basis of self reporting. Such a screener can never completely replace a medical consultation and a clinical examination of the patient. This is especially true for patients suffering from vertigo of multiple causes, e. g. PPV following organic vestibular disorders, or complicated BPPV. However, it can help to save time by allowing the examination to focus directly on the main symptoms. Furthermore, it may be a useful tool in epidemiological studies.

In the progress of building the diagnostic indices we noticed that in some cases characteristics individually had a relatively low sensitivity and specificity but that the sensitivity and specificity increased in combination with other characteristics.

### PPV

As expected, the construction of the diagnostic index for PPV turned out to be intricate, since the characteristics hardly had a sufficient sensitivity and specificity. In some cases an earlier specific vertigo disease (e.g. vestibular neuritis) can form the base of PPV [12], so the diagnostic index ought to include modified symptoms of the initial organic vertigo disease with the aid of variable combinations of characteristics.

### BPPV

While the clinical diagnosis of BPPV is quite straight forward, the symptoms of BPPV may be caused by different vertiginous diseases. Vestibular migraine imitates the symptoms of BPPV in some patients [13]. Furthermore, there seems to be a statistical connection between BPPV, MD and VM, without sufficient knowledge about the underlying pathophysiology [14]. Since they are typical BPPV symptoms, rotational vertigo and feeling "like on a roundabout" not surprisingly met the preselection criteria described in methods. Both were tested independently and combined as a composite variable (rotational vertigo OR roundabout) to resolve potential collinearity issues. Interestingly, including unsteadiness - which is a negative predictor of BPPV as can be seen from the negative sign in Table 2 - turned out to have better predictive power, than including rotational vertigo or feeling "like on a roundabout". Although the items used in our questionnaire were not specifically designed for BPPV, the sensitivity (0.875) and specificity (0.935) of the diagnostic index fit well with another study which reported a sensitivity of 0.88 and a specificity of 0.92 for recurrent attacks that lasted less than one minute and typical head movement that activates vertigo [15]. This combination of characteristics had a sensitivity of 0.38 in patients with BPPV in our study, since the sensitivity for vertigo attacks that lasted only seconds had a sensitivity of 0.38. Furthermore typical head movement as a trigger for vertigo had an equal sensitivity in patients with BPPV, PPV, MD and VM. The prevalence of rotational vertigo in BPPV patients was very similar (0.86 compared to 0.79 in our study).

### MD

Amongst other variables, the initial prediction model for MD contained the well-established triad rotational vertigo (sensitivity 0.64), ear noises and defective hearing. However, defective hearing was dropped during the selection process and thus does not appear in the final diagnostic index. Being aware of the triad, we tried a composite variable (defective hearing OR ear noises). However, omitting ear noises finally led to the better model. In comparison, a structured questionnaire on vertigo tested in a sample of 100 vertigo patients with a MD prevalence of 5% revealed a sensitivity, specificity and positive predictive value of 0.80, 0.97, and 0.57, respectively[16].

### VM

The combinations of symptoms appeared heterogeneous in patients with VM. Rotational vertigo had the highest sensitivity (0.79) but showed the same sensitivity in patients with BPPV and MD. This was already noticed in a former study [13]. According to the findings of Neuhauser and colleagues, the typical duration of attacks varies among patients and thus is not sufficiently specific [7]. The concomitant symptoms sweating/nausea/vomiting, which can also be found in the IHS criteria for migraine elevated the sensitivity and specificity of the diagnostic index. VM has already been characterized as "the chameleon of vertiginous diseases" due to the extreme variations of symptoms which may last from minutes to hours [17]. Nevertheless, the diagnostic index achieved a good sensitivity and specificity. In the ID migraine validation study among patients presenting for routine primary care appointments and reporting headaches in the past three months a subset of three questions was identified that revealed a sensitivity, specificity, and positive predictive value of 0.81, 0.75, and 0.93, respectively[2]. The much higher positive predictive value can be attributed to a higher prevalence of VM patients in this setting.

There are only a small number of other studies which have tried to establish a diagnostic questionnaire for vertigo. Most other studies have focused on the impact of vertigo on the quality of life or tried to estimate the subjective severity of vertigo. As there are established vertigo questionnaires designed for the purposes just described, it may be effective to extract diagnostic information from these tools. A study identified a subset of items from the dizziness handicap inventory (DHI) to detect BPPV in 373 patients referred to a tertiary center [18]. The resulting score was reported to have a maximum positive likelihood ratio of 2.29 as compared to 13.5 in the present study.

A study about the role of open-ended questionnaires conducted in 54 patients [19] suffering from vertigo, supported our methods: When questions had a number of possible answers, patients were more likely to report their symptoms in full.

Another study in 57 patients used a matrix classification based on type, episodic vs. persistent vertigo, and hearing loss to assign one of the diagnoses BPPV, MD, vestibular neuritis or labyrinthitis [20]. By comparison, the sensitivity and specificity of this tool were 0.50, 0.89 for BPPV, and 0.73, 0.81 for MD, respectively.

It should be noted, that we did not confine the patients to those given one of the four diagnoses investigated; 26% had other diagnoses. This reflects the clinical situation, where a patient complaining of vertigo is presented to the doctor, rather than a patient which is a priori known to have either PPV, BPPV, MD, or VM with the doctor only having to pick one out four possible diagnoses.

62 (32%) of the patients eligible could not be included in the analysis, most of them because of not returning the questionnaire. Keeping in mind, that the original questionnaire where the items for the diagnostic indices were embedded was 16 pages long, it is very likely to obtain better participation in future studies by shortening the questionnaire. A summary of the patients excluded has been published before ([3]http://www.biomedcentral.com/1471-2377/9/29, Table five). An overrepresentation of one of the four diagnoses of interest among these patients could give rise to concern that the questionnaire is not suitable to a specific group of patients. Compared to the patients included (PPV 53 (40.5%), BPPV 19 (14.5%), MD 11 (8.4%), VM 14 (10.7%)) such overrepresentation was not found, except for MD (12.9 vs. 8.4%, p = 0.43).

The limitations of our study include the small number of patients with MD (n = 11) and VM (n = 14), and the findings should therefore be considered preliminary. A test with a larger number of patients could help to prove whether the sensitivity and specificity of the screener hold. The diagnostic index was developed in patients referred to our outpatient clinic, which is a tertiary center for patients with vertigo and dizziness. In the majority of cases these patients suffer from chronic vertigo and were referred to our outpatient clinic after several consultations with medical specialists. This probably results in an overrepresentation of patients who were less easy to diagnose. The calculated sensitivity and specificity values may therefore be even better in unselected patients e. g. in a general practitioner's practice.

The advantage of our diagnostic indices is the development of one screening questionnaire with identical questions for four vertiginous diseases.

## Conclusions

We proposed a short screener, from which diagnostic scores for four prevalent vertiginous diseases can easily be calculated. Although cut-off points were provided the clinician or researcher may vary them to achieve better sensitivity or specificity as needed in the particular setting. The scores can also easily be converted to odds ratios (OR = e^score^) if desired. The test properties are promising and further validation in other populations is warranted.

## Competing interests

This study was conducted at the Department of Neurology, Ludwig-Maximilians-Universität Munich, Klinikum Großhadern, Marchioninistrasse 15, 81377 Munich, Germany, and was not sponsored by third parties.

Otmar Bayer reports no disclosures.

Jan Warninghoff reports no disclosures

Andreas Straube received payments for lectures and press conferences from Allergan Germany, Berlin Chemie Germany, MSD Germany, Pfizer Germany, Böhringer Ingelheim Germany

## Authors' contributions

OB did the statistical analysis, drafted the manuscript, and recruited patients. JW drafted the manuscript, and was responsible for the data management. AS conceived the study, contributed to the manuscript. All authors read and approved the final manuscript.

## Pre-publication history

The pre-publication history for this paper can be accessed here:

http://www.biomedcentral.com/1471-2377/10/98/prepub

## Supplementary Material

Additional file 1 **containing supplementary tables, examples of how to calculate the diagnostic scores, and the questionnaires used**. Click here for file
