# Correction:Diagnostic indices for vertiginous diseases

**DOI:** 10.1186/1471-2377-11-45

**Published:** 2011-04-14

**Authors:** Otmar Bayer, Jan-Christian Warninghoff, Andreas Straube

**Affiliations:** 1 Integrated center for research and treatment of vertigo, balance and ocular motor disorders Ludwig-Maximilians-University of Munich Heiglhofstr. 63, 81377 Munich, Germany; 2 Isar-Amper-Klinikum München-Ost, Vockestraße 72, 85540 Haar bei München, Germany; 3 Department of Neurology, University of Munich, Marchioninistraße 15, 81377 Munich, Germany

## 

We noticed after publication that there are errors in Table two (table 1 of this article) of our article [1], recently published in BMC Neurology.

**Table 1:** Table two corrected: Calculation of the diagnostic scores for PPV, BPPV, MD, and VM.

Question - Item/answer	PPV	BPPV	MD		VM
How does your vertigo occur?					
in attacks	0		3.77		
as persistent vertigo	2.22	-2.35			-1.79
as persistent vertigo with attacks	1.65				

What kind of your vertigo do you have?					
rotational vertigo	-1.48^1^				1.55^1^
unsteadiness		-3.26			
feeling of being in a lift	1.79	-3.06			
lightheadedness			-2.54		

How do you perceive the environment during vertigo?					
like on a roundabout	-1.48^1^				1.55^1^
like on a boat					
blurred		-3.42	-3.74		

How often do you have defective hearing?					
never					
occasionally					-1.14^2^
frequently					-1.14^2^
always					-1.14^2^

How often do you have ear noises?					
never					
occasionally					-1.14^2^
frequently					-1.14^2^
always		-3.03	5.42		-1.14^2^

How often do you have sweating/nausea/vomiting?					
never			0 = 0·0.98		
occasionally			0.98 = 1·0.98		
frequently		-1.21	1.95 = 2·0.98		2.82
always		-1.21	2.93 = 3·0.98		2.82

How often do you have drop seizures?					
never					
occasionally					
frequently	1.70	-2.95			
always		-2.95			

For answers marked in the questionnaire, the corresponding numbers are added up. Items marked with the same superscript number within one diagnosis are scored only once. E. g. the score for PPV of a patient reporting „rotational vertigo" or „perception of the environment like on a roundabout" (both marked by 1) will be diminished by 1.48 no matter whether this patient reports both or only one of these two symptoms. Patients are allowed to check only one answer per question, except for the 2nd and 3rd question.

Examples of calculated scores can be found in the Additional file 1.

Please find the corrections below:

1) A 2.82 is missing in column VM.

2) The superscripts in column BPPV are superfluous, as the last two questions allow only one answer each, as detailed in the caption.

These corrections are included in the spreadsheet files we offered upon request in the article.

## Pre-publication history

The pre-publication history for this paper can be accessed here:

http://www.biomedcentral.com/1471-2377/11/45/prepub
