# Supplementary material for: Diagnostic indices for vertiginous diseases
Source: BMC Neurol. 2010 Oct 25;10:98. doi: 10.1186/1471-2377-10-98 (PMC2987857; doi:10.1186/1471-2377-10-98)
Supplement: Additional file 1 — containing supplementary tables, examples of how to calculate the diagnostic scores, and the questionnaires used. [file 1471-2377-10-98-S1.PDF]

Additional file 1

Table S1: Spearman rank correlation coefficients for items used in the diagnostic indices.

	attacks vs. permanent vertigo	giddiness	unsteadiness	lift feeling	drowsiness	as in a roundabout	as in a boat	blurred	defective hearing	ear noises	sweating/nausea/vomiting	drop seizures
attacks vs. permanent vertigo	1	-0,30	0,21	0,05	0,02	-0,30	0,14	0,20	-0,02	0,03	-0,13	-0,07
rotational vertigo	-0,30	1,00	-0,50	-0,21	-0,10	0,70	-0,49	-0,16	0,20	0,04	0,15	0,26
unsteadiness	0,21	**-0,50**	1,00	0,00	-0,06	-0,35	0,52	-0,25	-0,09	-0,01	-0,11	-0,23
lift feeling	0,05	-0,21	0,00	1,00	0,00	-0,23	0,24	0,07	-0,20	-0,08	-0,11	-0,13
lightheadedness	0,02	-0,10	-0,06	0,00	1,00	-0,16	0,14	0,33	-0,04	0,03	0,05	0,03
like on a roundabout	-0,30	**0,70**	-0,35	-0,23	-0,16	1,00	-0,68	-0,20	0,13	-0,16	0,16	0,17
like on a boat	0,14	-0,49	**0,52**	0,24	0,14	**-0,68**	1,00	-0,24	-0,13	0,12	-0,15	-0,13
blurred	0,20	-0,16	-0,25	0,07	0,33	-0,20	-0,24	1,00	0,12	0,06	-0,02	0,06
defective hearing	-0,02	0,20	-0,09	-0,20	-0,04	0,13	-0,13	0,12	1,00	0,58	0,13	0,45
ear noises	0,03	0,04	-0,01	-0,08	0,03	-0,16	0,12	0,06	**0,58**	1,00	0,11	0,24
sweating/nausea/vomiting	-0,13	0,15	-0,11	-0,11	0,05	0,16	-0,15	-0,02	0,13	0,11	1,00	0,27
drop seizures	-0,07	0,26	-0,23	-0,13	0,03	0,17	-0,13	0,06	0,45	0,24	0,27	1,00

Bold print indicates correlations equal to or stronger than 0.5

Contingency tables for complete cases at the suggested cut-off points
These were included for compliance with STARD. Please note, that cut-off points may be varied as explained in the Discussion.

PPV	diagnosis		
test	POSITIVE	NEGATIVE	Sum
POSITIVE	28	10	38
NEGATIVE	10	53	63
Sum	38	63	101

BPPV	diagnosis		
test	POSITIVE	NEGATIVE	Sum
POSITIVE	14	5	19
NEGATIVE	2	72	74
Sum	16	77	93

MD	diagnosis		
test	POSITIVE	NEGATIVE	Sum
POSITIVE	9	11	20
NEGATIVE	0	76	76
Sum	9	87	96

VM	diagnosis		
test	POSITIVE	NEGATIVE	Sum
POSITIVE	12	14	26
NEGATIVE	1	72	73
Sum	13	86	99

Questions used to build the diagnostic indices

How does your vertigo occur?	in attacks ☐	as persistent vertigo ☐	as persistent vertigo with attacks ☐	
What kind of vertigo do you have?	rotational vertigo ☐	unsteadiness ☐	feeling of being in a lift ☐	lightheaded ness ☐
How do you perceive the environment during vertigo?	like on a roundabout ☐	like on a boat ☐	very blurred ☐	
How often do you have defective hearing?	never ☐	occasionally ☐	frequently ☐	always ☐
How often do you have ear noises?	never ☐	occasionally ☐	frequently ☐	always ☐
How often do you have sweating/nausea/vomiting?	never ☐	occasionally ☐	frequently ☐	always ☐
How often do you have drop seizures?	never ☐	occasionally ☐	frequently ☐	always ☐

Examples of calculated scores

Patient marked the following answers in the questionnaire:
in attacks, rotational vertigo, feeling of being in a lift, like on a roundabout, occasionally, always, occasionally, never. Using the values from Table 2 and the cut-off values provided in Results the
Score for BPPV = 0 + 0 - 3.06 + 0 + 0 - 3.03 + 0 + 0 = -6.09 < -1.22 → BPPV not suspected
Score for MD = 3.77 + 0 + 0 + 0 + 0 + 5.42 + 0.98 + 0 = 10.17 > 6.70 → MD suspected

The authors are happy to provide spreadsheets that calculate the scores for all four diagnoses automatically for use with Microsoft Excel or in OpenDocument format for use with various spreadsheet programs, e. g. OpenOffice (free download from http://download.openoffice.org).

Questions used to build the diagnostic indices - German version

Wie tritt Ihr Schwindel auf?	als Attacke ☐	als Dauerschwindel ☐	als Dauerschwindel mit Attacken ☐	
Wie äußert sich Ihr Schwindel?	als Drehschwindel ☐	als Schwankschwindel ☐	als Liftgefühl ☐	als Benommenheit ☐
Wie nehmen Sie ihre Umgebung während des Schwindels wahr?	wie im Karussell ☐	wie im Boot ☐	ganz verschwommen ☐	
Wie oft haben Sie Hörstörungen?	nie ☐	selten ☐	häufig ☐	immer ☐
Wie oft haben Sie Ohrgeräusche?	nie ☐	selten ☐	häufig ☐	immer ☐
Wie oft haben Sie Schweißausbrüche/ Übelkeit/Erbrechen?	nie ☐	selten ☐	häufig ☐	immer ☐
Wie oft haben Sie Sturzanfälle?	nie ☐	selten ☐	häufig ☐	immer ☐
